# Functionalized nanoparticles with targeted antibody to enhance imaging of breast cancer in vivo

**DOI:** 10.1186/s12951-020-00695-2

**Published:** 2020-09-18

**Authors:** Jesse S. Chen, Jingwen Chen, Somnath Bhattacharjee, Zhengyi Cao, Han Wang, Scott D. Swanson, Hong Zong, James R. Baker, Su He Wang

**Affiliations:** 1 Department of Internal Medicine, Division of Allergy, Michigan Nanotechnology Institute for Medicine and Biological Sciences, University of Michigan, 109 Zina Pitcher Place, Ann Arbor, MI 48109 USA; 2 Department of Radiology, Shanghai General Hospital, Shanghai Jiao Tong University School of Medicine, Shanghai, People’s Republic of China; 3 Department of Radiology, University of Michigan, 1500 East Medical Center Drive, Ann Arbor, MI 48109 USA

**Keywords:** Targeting, Nanoparticles, Bi-modal imaging, Computerized tomography, Magnetic resonance imaging, HER-2

## Abstract

**Background:**

Targeted contrast nanoparticles for breast tumor imaging facilitates early detection and improves treatment efficacy of breast cancer. This manuscript reports the development of an epidermal growth factor receptor-2 (HER-2) specific, bi-modal, dendrimer conjugate to enhance computed tomography (CT) and magnetic resonance imaging (MRI) of HER-2-positive breast cancer. This material employs generation 5 poly(amidoamine) dendrimers, encapsulated gold nanoparticles, chelated gadolinium, and anti-human HER-2 antibody to produce the nanoparticle contrast agent.

**Results:**

Testing in two mouse tumor models confirms this contrast agent’s ability to image HER-2 positive tumors. Intravenous injection of this nanoparticle in mice bearing HER-2 positive mammary tumors significantly enhances MRI signal intensity by ~ 20% and improves CT resolution and contrast by two-fold. Results by flow cytometry and confocal microscopy validate the specific targeting of the conjugate and its internalization in human HER-2 positive cells.

**Conclusion:**

These results demonstrate that this nanoparticle conjugate can efficiently target and image HER-2 positive tumors in vivo and provide a basis for the development of this diagnostic tool for early detection, metastatic assessment and therapeutic monitoring of HER-2 positive cancers. 
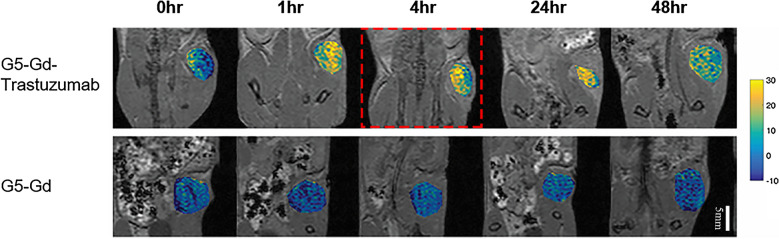

## Background

Breast cancer is the second most common cancer diagnosed for women in the United States with more than 268,600 new cases estimated for 2019; it is also the second highest cause of cancer-related death in women. [1] While a great effort has been made to improve breast cancer management, a remaining challenge is the development of imaging agents that can enhance early diagnosis. This is important since the early detection of breast cancer is vital to improve the treatment outcome and patient survival rates. While the primary method for diagnosis of breast cancer is x-ray mammography, over the past 20 years, magnetic resonance imaging (MRI) methods of dynamic contrast enhancement and 3D lesion characterization have increased the sensitivity and specificity of breast cancer diagnosis; despite this, these methods have not eliminated the need for biopsy [2–4]. Dynamic contrast enhanced MRI has been indicated to monitor certain groups of women at high risk of developing breast cancer [5]. MRI has the benefits of providing the correct contrast in soft tissue imaging as well as tomographic images without the need for radiation [6]. In comparison to ultrasound or mammography, MRI provides more accurate early-stage tumor imaging as well as detection of cancer invasiveness [7, 8]. However, the sensitivity and specificity of the information conveyed by MRI depend predominantly on the contrast agent used. Gadolinium (Gd), particularly when chelated with 1,4,7,10-tetraazacyclododecane-1,4,7,10-tetraacetic acid (DOTA), is typically used for MRI due in part to the relaxation time and ease of visualization [9]. Computerized tomography (CT) has also been improved through the use of gold nanoparticles (AuNPs). These nanoparticles offer augmented resolution and contrast, compared to iodine-based agents, because of their higher X-ray absorption coefficient. Additionally, in combination with a particle platform, such as dendrimers, AuNPs have shown an increase in biocompatibility, stability, and half-life decay [10–12]. However, a downside with both CT and MRI methods is that low target specificity may require additional imaging and procedural steps that are problematic and overly invasive [7]. To improve the specificity, several cancer cell markers, such as the folic acid receptor and transferrin receptor, have been targeted to deliver contrast agents [13, 14].

Breast cancer is a highly heterogeneous disease, and 20–30% of breast cancers are characterized by the overexpression of human epidermal growth factor receptor 2 (HER-2) [15]. This overexpression can lead to enhancement of cell growth and proliferation. HER-2 positive breast cancer is often more aggressive and resistant to conventional chemotherapy [16–20]. Targeted therapy with humanized monoclonal anti-HER-2-antibody (Trastuzumab) has become a mainstay treatment of HER-2 positive breast cancer [21]. However, while drug conjugates with Trastuzumab have shown clinical utility, [22] imaging with this agent has not advanced. One approach to developing an active targeting, receptor-based imaging agent combined with therapeutic delivery is the use of nanoparticles. As a nanoparticle, the dendrimer has a highly-branched structure with large numbers of functional ending groups [23]. Along with other star polymers, dendrimers are a class of molecules that have shown higher stability of conjugated materials, as well as better biocompatibility, when compared to linear-shaped polymers [24]. Polyamidoamine (PAMAM) dendrimers are suitable for many material sciences and biotechnology applications due to a narrow poly-dispersity and multivalent conjugation potential. Studies have demonstrated that PAMAM dendrimers can be covalently coupled with biological molecules such as chemotherapeutic drugs, [13] DNA, [25] antibodies, [26, 27] and MRI contrast agents [28]. Additionally, it has been demonstrated that capping most surface amino groups in the conjugates can minimize cytotoxicity [29]. Importantly, our prior in vitro studies have shown the dendrimer, coupled with anti-HER-2 antibody, has potential use in both imaging and therapeutic applications of HER-2-positive breast cancer [10, 30].

Herein, we reported the utilization of the Generation 5 PAMAM dendrimer conjugated with gold nanoparticles (G5-AuNP), DOTA-Gd, and Trastuzumab for subsequent MRI and CT imaging of breast cancer tumors in vivo. This study has demonstrated that the Trastuzumab-guided dual-imaging conjugate can specifically target and enhance both MRI signal and CT resolution of HER-2 positive breast tumors.

## Results

### Signal intensity enhanced with G5-AuNP-Gd-Trastuzumab for CT scanning and MRI of A549-inoculated BALB/c nude mice

**Fig. 1:**
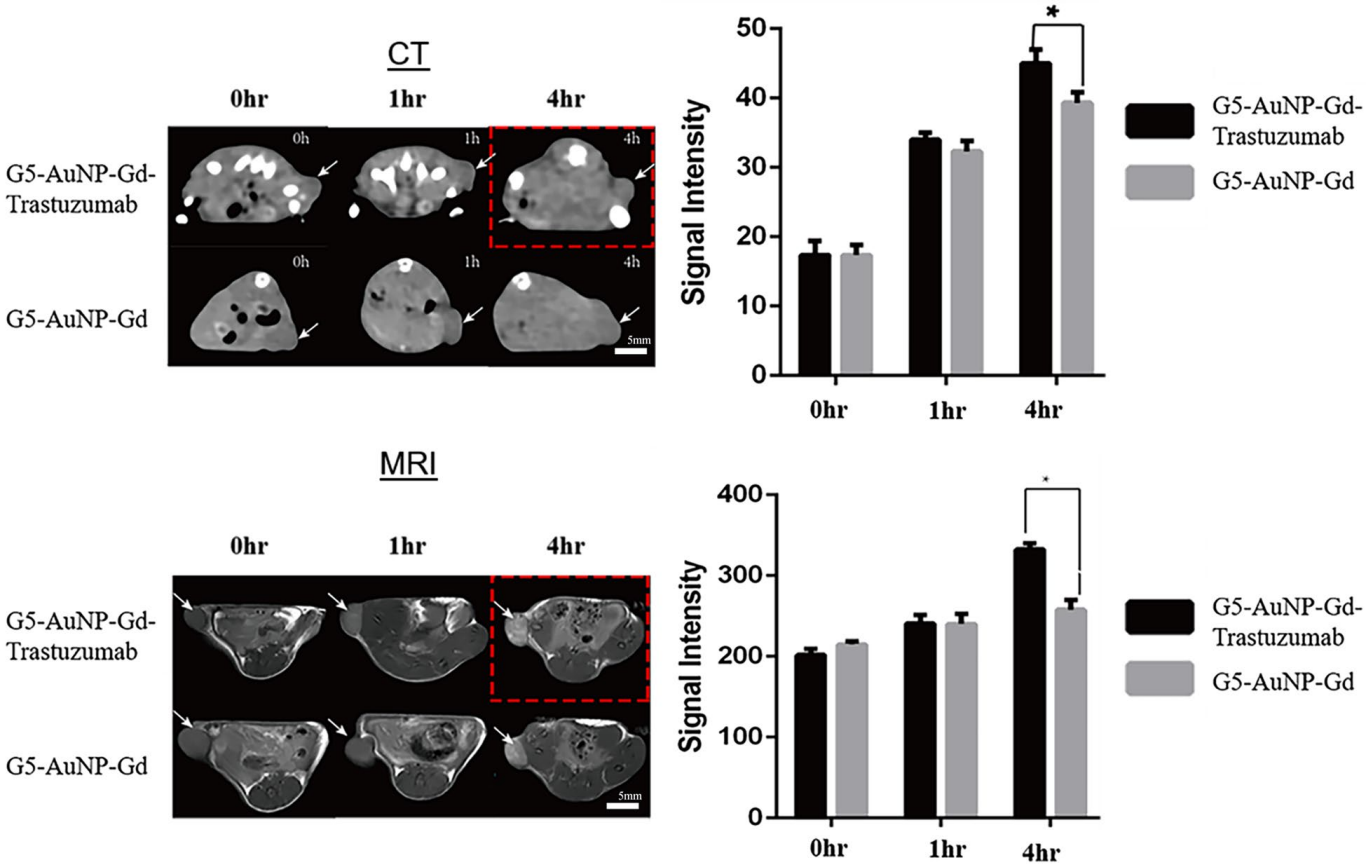
CT & MRI results from BALB/c nude mouse model. Imaging results from tumor-bearing mice inoculated with A549 cells. CT scans (top) show that with G5-AuNP-Gd-Trastuzumab (red box), there is a significant difference in signal intensity when compared to non-targeted G5-AuNP-Gd at 4 h after conjugate administration. MRI (showed in the bottom panel), also demonstrates a significant difference between G5-AuNP-Gd-Trastuzumab (red box) and G5-AuNP-Gd at the 4 h time point, *P* = 0.05. SEM was used for error bars, n = 6

Based on the binding data obtained from our previous in vitro work, [10, 30] the dual-imaging targeted conjugate G5-AuNP-Gd-Trastuzumab was tested for in vivo imaging efficacy in tumor-bearing mice inoculated with HER-2 positive A549 cells. CT scans and MRI were taken over a 48 h period after conjugate administration. When examining CT scan results (Fig. 1), there was a 13% increase in signal intensity (*p* = 0.05) at 4 h after conjugate administration, with G5-AuNP-Gd-Trastuzumab compared to G5-AuNP-Gd. Additionally, G5-AuNP-Gd-Trastuzumab significantly increased signal intensity (*p* = 0.05) at 8 h as well (data not shown). MRI results (Fig. 1) revealed that, similar to the CT data, G5-AuNP-Gd-Trastuzumab significantly increased signal intensity at the 4 h time point, by 30% (*p* = 0.05), compared to non-targeted G5-AuNP-Gd.

### G5-Gd-Trastuzumab-AF647 demonstrates HER-2 targeted in vitro binding and internalization, with minimal cytotoxicity in HER-2 expressing transfected E0771-E2 cells

**Fig. 2:**
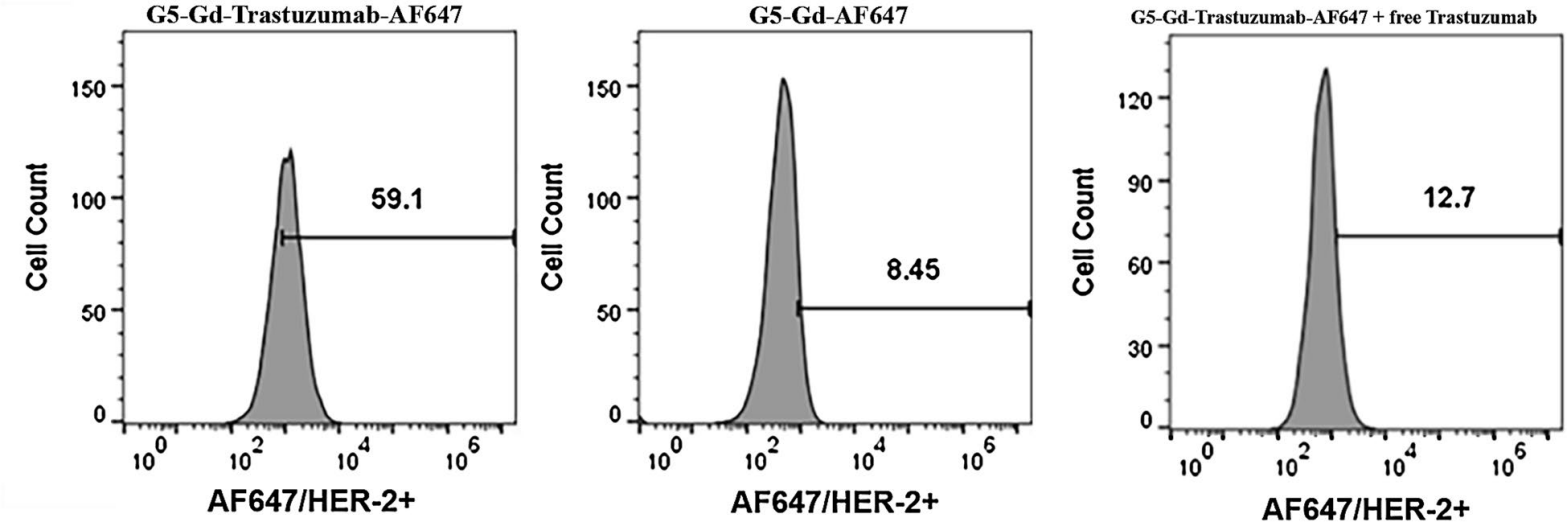
Binding and internalization in E0771-E2 cells with either G5-Gd-Trastuzumab-AF647 or G5-Gd-AF647. E0771-E2 cells were incubated with either targeted or control conjugates (conjugate used is labeled above each histogram) and analyzed by flow cytometry. Histograms are presented of E0771-E2 cells incubated with G5-Gd-Trastuzumab-AF647 (left panel), G5-Gd-AF647 (middle panel) or G5-Gd-Trastuzumab-AF647 in an excess of free Trastuzumab (right panel). The histograms show specific uptake of HER-2 positive cells (59.1%) as compared to non-targeted material (8.5%) and targeted material competing with free antibody (12.7%) as detected via the AF647 staining (x-axis). Gates were defined from unstained E0771-E2 cells

After testing of the dual-imaging conjugate in mice inoculated with A549 cells, the platform was further examined in a mouse using HER-2 expressing syngeneic tumor cells. The goal of further testing was to explore the pharmacokinetics of the conjugate, with an emphasis on additional evaluation of the conjugate’s MRI enhancement in a more relevant tumor model. To confirm HER-2 specificity binding to the E0771-E2 cells, flow cytometry was employed to measure the conjugate binding specificity. As Shown in Fig. 2, E0771-E2 cells incubated with G5-Gd-Trastuzumab-AF647 showed a ~ 54% increase in fluorescent intensity from baseline, compared to non-targeted G5-Gd-AF647 (~ 4% increase). HER-2 negative E0771 cells showed no increase compared to untreated cells when incubated with either conjugate (data not shown).

Additionally, to further confirm HER-2 specific binding with G5-Gd-Trastuzumab-AF647, free Trastuzumab was added as a competitor and binding of the conjugate was again determined by flow cytometry. The results showed that after pre-incubation with excess levels of Trastuzumab, the targeted conjugate only increased fluorescent intensity in E0771-E2 cells by 8% compared to baseline (Fig. 2). This showed a sixfold decrease in fluorescent intensity compared with G5-Gd-Trastuzumab-AF647 in the absence of Trastuzumab pretreatment.

**Fig. 3:**
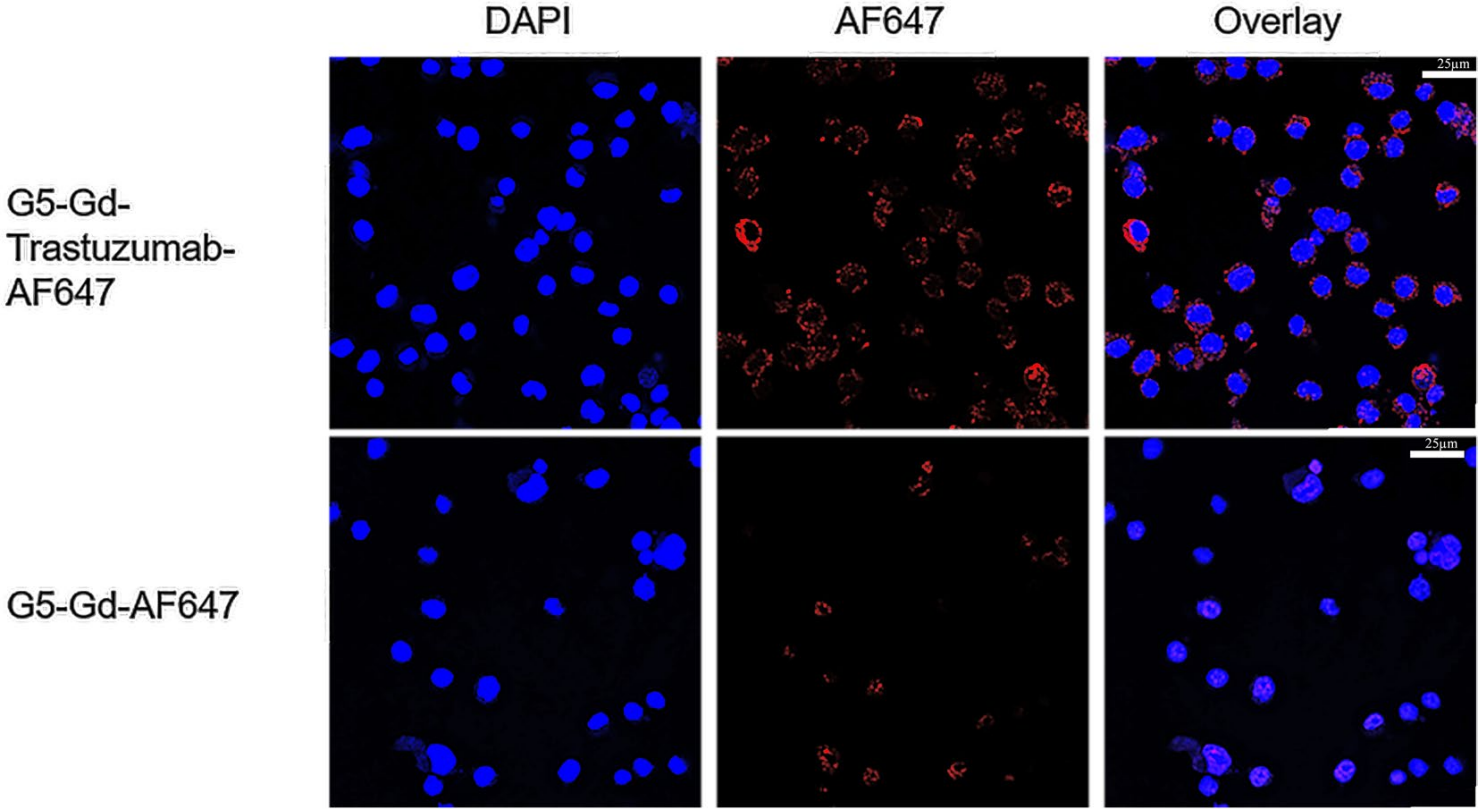
The internalization of G5-Gd-AF647 & G5-Gd-Trastuzumab-AF647 into E0771-E2 cells after 18 h incubation. Top panel shows E0771-E2 cells incubated with G5-Gd-Trastuzumab-AF647 and DAPI, overlaid. Bottom panel shows cells incubated with G5-Gd-AF647. First column shows the DAPI staining (blue), followed by the AF647 channel (red) in the second column, and then finally a merged view in the third column. The AF647 fluorescence shows that not only is G5-Gd-Trastuzumab-AF647 binds to targeted HER-2, but also there is also evidence of internalization into the cell

Confocal microscopy was used to analyze the internalization of G5-Gd-Trastuzumab-AF647 conjugate. As shown in Fig. 3, there were significant increases in the number of E0771-E2 cells with positive cytoplasmic fluorescence at 18 h when incubated with G5-Gd-Trastuzumab-AF647 (92% of cells) compared with non-targeted G5-Gd-AF647 (48% of cells). This is in accordance with increases in AF647-positive cells incubated at various time points with G5-Gd-Trastuzumab-AF647 (data not shown). The in vitro cytotoxicity of G5-Gd-Trastuzumab-AF647 was examined via XTT assay. After a 48 h incubation, E0771 and E0771-E2 cells showed similar levels of viability (~ 95% viable cells) with or without either conjugate treatment, suggesting that for antibody-dependent cellular cytotoxicity, the antibody conjugates were not inherently cytotoxic to the E0771-E2 cells.

### Kinetics of G5-Gd-Trastuzumab MRI signal intensity enhancement in E0771-E2-inoculated HER-2 transgenic mice

**Fig. 4:**
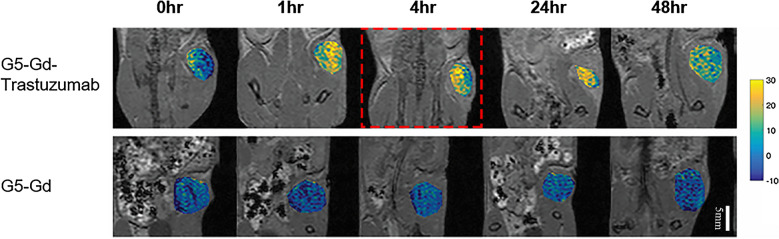
MRI of tumors from mice injected with either G5-Gd-Trastuzumab or G5-Gd nanoparticles. Images were obtained prior to injection of contrast agents (base image) and at 1, 4, 24 and 48 h post injection. The signal intensity in each image was normalized to the muscle tissue signal to account for signal variations due to animal position in the MRI probe. Imaging shows increased uptake of targeted particles that persisted for 48 h

**Fig. 5:**
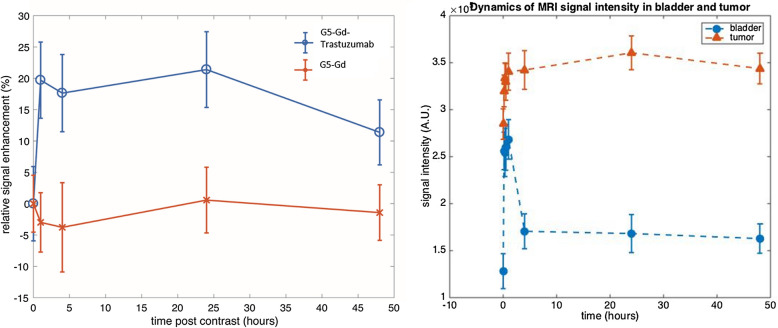
MRI signal enhancement in tumors from mice injected with G5-Gd-Trastuzumab or G5-Gd. Quantitation of signal enhancement in mice receiving G5-Gd-Trastuzumab (circles) vs. G5-Gd (Xs) at 1, 4, 24 and 48 h post injection (left panel). Difference between the two treatments is statistically significant (*P* < 0.001) at all the measured time points. Results are shown as mean ± standard deviation of the pixel intensities in the tumor. Biodistribution time course shows persistence of signal in the tumor as compared to urinary excretion (bladder imaging, right panel). SEM was used for error bars, n = 10

**Fig. 6:**
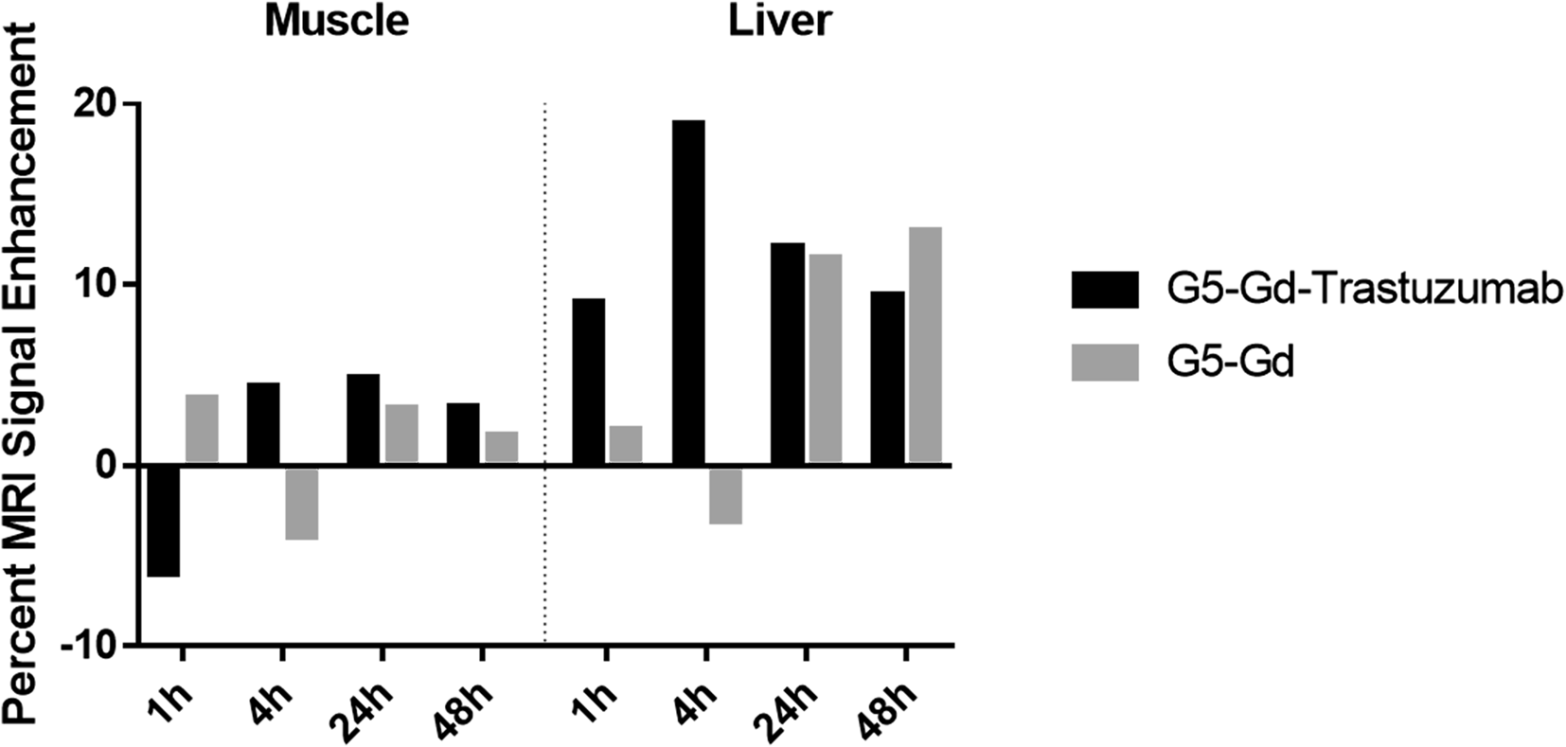
Biodistribution study of mice injected with either G5-Gd-Trastuzumab or G5-Gd nanoparticles. MRI signal enhancement was measured in muscle (left) and liver (right). There is minimal change in the MRI signal over the course of the study in muscle. Liver showed initial enhancement for G5-Gd-Trastuzumab and accumulation of both conjugates after 1 and 2 days post injection

The pharmacokinetics of the targeted conjugate in vivo was examined using MRI in HER-2 transgenic mice, inoculated with HER-2 positive E0771-E2 cells. Tumor-bearing mice received either G5-Gd-Trastuzumab or G5-Gd, via the tail vein injection. Images and signaling intensity were acquired with MRI at 0, 1, 4, 24 and 48 h post conjugate administration (Figs. 4 and 5). To visualize increases in signal intensity, the pixel value of the baseline tumor signal was first normalized to 100%. The percent MRI enhancement was calculated as 100 × [S(t)-S(base)]/S(base)]. Figure 5 shows that G5-Gd-Trastuzumab had enhanced MRI tumor signal intensity by more than 20% when compared to G5-Gd enhancement at 4 h after injection. Additionally, the enhanced tumor signals from G5-Gd-Trastuzumab were maintained even at 48 h after injection as compared to G5-Gd (Fig. 4). While the biodistribution analysis of non-target organs revealed some non-specific muscle, liver, and bladder accumulation of both conjugates (Figs. 5 and 6), only the targeted conjugate accumulated in the tumors.

## Discussion

Breast cancer is known to be a heterogeneous disease based on multiple genetic abnormalities. One such factor, the overexpression of HER-2, is observed in approximately 20–30% of all breast cancers and has been associated with therapeutic resistance, early relapse, and poor prognosis [15–18]. HER-2 targeted treatment strategies require the identification of the expression of this molecule, usually by immunological histologic techniques on tumor samples. While this approach has been effective, it is invasive and can be associated with sampling error. It also does not give a perspective of the overall tumor burden or distribution of HER-2 positive cells. Therefore, HER-2 tumor imaging as an early-stage diagnostic tool would be a useful option in addition to more invasive techniques.

The goal of our current study is to document the feasibility of HER-2 specific imaging using a dual imaging agent based on a dendrimer, nanoparticle platform. We designed and tested a bi-modal CT/MRI imaging agent, G5-AuNP-Gd-Trastuzumab, in vitro, with both HER-2 positive and negative cells, to confirm both binding and internalization via flow cytometry and confocal microscopy. Importantly, we successfully demonstrated enhanced and specific HER-2 based uptake in CT imaging and MRI. These results were confirmed in tumor-bearing mice inoculated with A549 cells, enhancing both CT and MRI signal intensities by two-fold. Additionally, we investigated the pharmacokinetics of the conjugate for MRI in immunocompetent HER-2 transgenic mice inoculated with HER-2 transfected E0771-E2 cells. The conjugate was able to enhance MRI tumor signal intensity by ~ 21%, with signal improvements that persisted for 24 h after injection.

MRI and CT have unique capabilities vs. mammography and ultrasound as breast cancer imaging techniques. For instance, CT has been utilized to document distant metastasis, [31] cancer staging, [32] and segmentation of tissues for possible tumor detection [33]. MRI has the capability of detecting lesions that cannot be seen with mammography [31]. Notably, one of the main benefits of the multi-modal imaging platform compared to single method imaging is the complementary aspect of the imaging agents. For example, a bi-modal CT/MRI conjugate can improve the imaging precision and the contrast enhancement compared to any singular imaging agent as shown with lung cancer [34]. Adding HER-2 targeting enhancement to these capabilities will substantially improve the utility of CT/MRI. In addition, while published reports have documented ultrasound/MRI targeted imaging of HER-2 positive breast cancer cells, [35, 36] we believe that this novel conjugate provides a more therapeutically meaningful option by combining CT and MRI targeted imaging for the early detection of HER-2 positive breast cancer.

While most studies perform imaging 2–4 h after agent administration, [34, 37] we have explored the pharmacokinetics of our conjugate for longer periods of time. From these results, specifically by the MRI data from our transgenic HER-2 mouse model, it shows persistence of signal enhancement out to 24 h after initial injection, similar to earlier time points at 4 and 8 h after injection for both CT and MRI. Though imaging agents have proved to be an invaluable tool, circulation time and biodistribution of these agents are concerns [38, 39]. This is because increasing circulation times and extending the signal enhancement window, usually results in imaging agents being retained in untargeted organs. Our results differ in two important ways. The MRI results from our conjugate showed clear HER-2 targeting as compared to the low tumor specificity of current MRI agents [31]. Also, while there are small amounts of the conjugate retained within non-targeted organs, the differences in this material were not statistically significant from baseline levels, suggesting they were extremely small.

While the focus for this conjugate surrounds the concept of an early detection imaging agent, there are additional applications that this dual CT/MRI functionality could prove useful in for a clinical setting. For example, MRI prior to neo-adjuvant chemotherapy has been shown to clarify whether the treatment will increase the likelihood of pathologic complete response [40–42]. Additionally, a recent review describes the potential utilization of both MRI and CT techniques in order to image and diagnose breast cancer brain metastases; [37] this may be of particular interest for a HER-2 targeted imaging conjugate since HER-2 positive cancers are more aggressive [22].

## Conclusions

In summary, these studies show the successful, in vivo validation of a functional CT/MRI, Trastuzumab targeted, dual-imaging conjugate in mouse models. This included documenting the specificity and pharmacokinetics of the conjugate in MRI in immunocompetent, HER-2 transgenic mice inoculated with HER-2 transfected E0771 cells. We also demonstrated the specificity of the conjugate in vitro, using HER-2 positive and negative cells, to confirm both binding and internalization using flow cytometry and confocal microscopy. This work indicates that the bi-modal nanoparticle imaging conjugate can target and enhance both MRI signal and CT resolution in HER-2 positive tumors, providing potential utility for the use of this material in the early detection and identification of HER-2 expression in human tumors.

## Methods

### Materials

All solvents and chemicals were of reagent grade quality, purchased from Sigma-Aldrich (St. Louis, MO), and used without further purification unless otherwise noted. G5 PAMAM dendrimer was purchased from Dendritech (Midland, MI). Monomethyl-PEG^11^-NHS (PEG-NHS) ester was purchased from ChemPep Inc. 3-(4-(2-azidoethoxy) phenyl) propanoic acid was used from previous studies. 2,2′,2′′-(10-(2-((2,5-dioxopyrrolidin-1-yl)oxy)-2-oxoethyl)-1,4,7,10-tetraazacyclododecane-1,4,7-triyl) triacetic acid (DOTA-NHS) was purchased from Macrocyclics (Dallas, TX). AF647-azide was purchased from Life Technologies of Thermo Fisher Scientific (Grand Island, NY). Trastuzumab was obtained from Genetech (San Francisco, CA).

### Synthesis and characterization of conjugates

G5-AuNP-Gd-Trastuzumab was synthesized and analyzed as previously published [10]. This conjugate was used to evaluate tumor signal intensity by both MRI and CT in A549-inoculated SCID mouse model. These conjugates were modified to G5-Gd-Trastuzumab and G5-Gd-Trastuzumab-AF647 for HER-2 transgenic mice and in vitro studies as follows:

First, G5-PEG-Alkyne-DOTA-NHAc was synthesized following published procedures [10]. Then G5-PEG-Alkyne-DOTA-Gd-NHAc was produced by mixing aqueous Gd(NO_3_)_3_ with G5-PEG-Alkyne-DOTA-NHAc. Final conjugate G5-Gd-Trastuzumab was synthesized by copper-catalyzed click reaction using G5-PEG-Alkyne-DOTA-NHAc and Trastuzumab-azide (Additional file 1. Fig. S1).

For in vitro study, G5-Gd-Trastuzumab-AF647 was generated in s similar fashion using G5-Gd-Trastuzumab and AF647-azide (Additional file 2. Fig. S2).

Characterization, such as MADLI, UPLC, and 1H NMR, of materials used is provided in Additional file 3. Fig. S3 and Additional file 4. Fig. S4, while additional characterization of the conjugate platform, such as dynamic light scattering and zeta potential, has been previously published. [10].

### Determination of conjugate cytotoxicity

E0771 mouse mammary tumor cells were derived from mammary cancer of a C57BL/6 mouse and stably transfected with full-length human HER-2 cDNA to overexpress the human HER-2 antigen (E0771-E2) [43]. Cell viability of E0771-E2 cells, when incubated with various concentrations of G5-Gd-Trastuzumab, was determined by XTT assay. E0771-E2 cells were planted in a 96-well tissue culture plate and incubated with conjugates for 48 h. XTT reagents (Sigma-Aldrich; St. Louis, MO) were added to each well prior to absorbance measurements via Synergy HT microplate reader (BioTek Instruments; Winooski, VT). The difference in optical densities at 690 nm subtracted from 492 nm was used to calculate cell viability percentages.

### Tumor models for CT and MRI

Two mouse tumor models were used in this study: one was established in immunodeficient BALB/c nude mice inoculated with A549 cells and the other in non-immunodeficient C57BL/6 mice implanted with E0771-E2 cells. Both A549 cells and E0771-E2 cells express HER-2 [10].

For the BALB/c nude model, 12 mice (4–6 weeks old) were purchased from Shanghai SLAC Laboratory Animal Co., Ltd., Shanghai, China. A549 cells (1 × 10^6^ cells) in 100 μl PBS were subcutaneously implanted into the right flank of the mice. When the tumor nodules reached to a volume of 75 mm^3^, those mice were injected with 100 µl of the conjugate (5 µg/µl) intravenously for in vivo imaging. The mice were measured using a CT imaging system (SOMATOM Definition Flash, SIEMENS, Germany) with 80 kV, 50 mA and a slice thickness of 0.625 mm. CT signal intensities in the region of interest were acquired pre-injection and at time points of 1, 4, 8 and 12 h post-injection. The A549 tumor-bearing mice were also acquired on a 3.0 T MRI system (Discovery MR750, GE Healthcare, Knox, IN). The parameters of MRI were set as follows: TR = 400 ms, TE = 12.2 ms, NEX = 4.00, matrix = 256 × 256, slice thickness = 2 mm, slice space = 0.8 mm, and FOV = 12 cm. T_1_ weighted images were obtained both before and after injection at time points of 1, 4, 24 and 48 h post-injection.

For the HER-2 transgenic model, 1 × 10^6^ E0771-E2 cells in 100 µl of PBS were injected subcutaneously into the flank of 8, 6-week old mice. [43] The cells were allowed to grow until tumors reached an approximate size of 0.5 − 1 cm in diameter. Tumor growth was measured twice per week. Tumor-bearing mice received 100 µl of the conjugate (5 µg/µl) via the tail vein injection. MRI was acquired with a 2 T Varian Unity/Inova MRI small animal imaging system equipped with Acustar S-180 gradients. The initial baseline MRI was obtained from each mouse, and mice were imaged at 1, 4, 24 and 48 h after the administration of conjugates. Each image was acquired with a 3D gradient echo pulse sequence with a TR of 20 ms, TE of 4 ms, flip angle of 20° and isotropic voxel size of 200 microns.

Following collection, data were processed in Matlab (The Mathworks, Natick, MA). The 3D k-space data were Fourier transformed to generate 3D images. The region of the tumor was identified on the images and a 3D ellipsoidal region of interest was drawn around the tumor. A similar 3D ellipsoidal region was drawn in muscles of the hind leg. MRI signal intensities from each of these regions were measured. To reduce the variation in MRI probe tuning or animal positions that may occur from scan to scan, the MRI tumor signal intensity was normalized with the muscle signal intensity. The normalized histogram of the pixel intensities of the tumor area was computed by dividing the tumor data by the average muscle value. MRI signal enhancement was calculated based on the increase of signals in the tumor generated by the conjugates relative to the baseline (non-contrast) image.

### In vitro binding and internalization assays for E0771-E2 cells

The specific conjugate binding to cell surface HER-2 receptors was tested with flow cytometry. Prior to staining, cells were blocked with 1% bovine serum albumin (BSA). After samples were incubated with G5-Gd-AF647 or G5-Gd-Trastuzumab-AF647 for 1 h, they were analyzed with an Accuri C6 Flow Cytometer (BD Biosciences, San Jose, CA). In addition, conjugates were tested in competition with anti-HER-2 (10ug/ml Trastuzumab, Genetech). Cells were incubated with Trastuzumab for 30 min, washed once, and treated with either G5-Gd-AF647 or G5-Gd-Trastuzumab-AF647. Then, samples are analyzed with the Accuri C6 Flow Cytometer and FlowJo software.

Testing for conjugate internalization was also performed. Cells were plated into a 6-well confocal plate and incubated with G5-Gd-AF647 or G5-Gd-Trastuzumab-AF647 for up to 18 h. After incubation, cells were fixed with 2% paraformaldehyde. Samples are mounted with ProLong Gold Antifade Reagent with DAPI (Life Technologies). Imaging was done with a Leica Inverted SP5 Microscope.

### Statistical analysis

Data were analyzed using a two-tailed unpaired t-test with Welch’s correction. *P* values of less than 0.05 were considered statistically significant.

## Supplementary information

**Additional file 1:** **Figure S1.** Preparation of G5-Gd-Trastuzumab (7) and G5-Gd (5).

**Additional file 2:** **Figure S2.** Preparation of G5-Gd-Trastuzumab-AF647 (9) and G5-Gd-AF647 (8), from G5-Gd-Trastuzumab (7) and G5-Gd (5).

**Additional file 3:** **Figure S3.** MALDI, UPLC, and synthesis information.

**Additional file 4:** **Figure S4.** Size exclusion chromatography and NMR for G5-Gd.

## Data Availability

All data generated or analyzed during this are included in this published article [and its supplementary information files].

## Abbreviations

CT: Computed tomography
MRI: Magnetic resonance imaging
Gd: Gadolinium
DOTA: 1,4,7,10-Tetraazacyclododecane-1,4,7,10-tetraacetic acid
AuNP: Gold nanoparticle
HER-2: Human epidermal growth factor receptor-2
PAMAM: Polyamidoamine; BSA, bovine serum albumin
