# Supplementary figures and images for: Functionalized nanoparticles with targeted antibody to enhance imaging of breast cancer in vivo
Source: J Nanobiotechnology. 2020 Sep 18;18:135. doi: 10.1186/s12951-020-00695-2 (PMC7501678; doi:10.1186/s12951-020-00695-2)

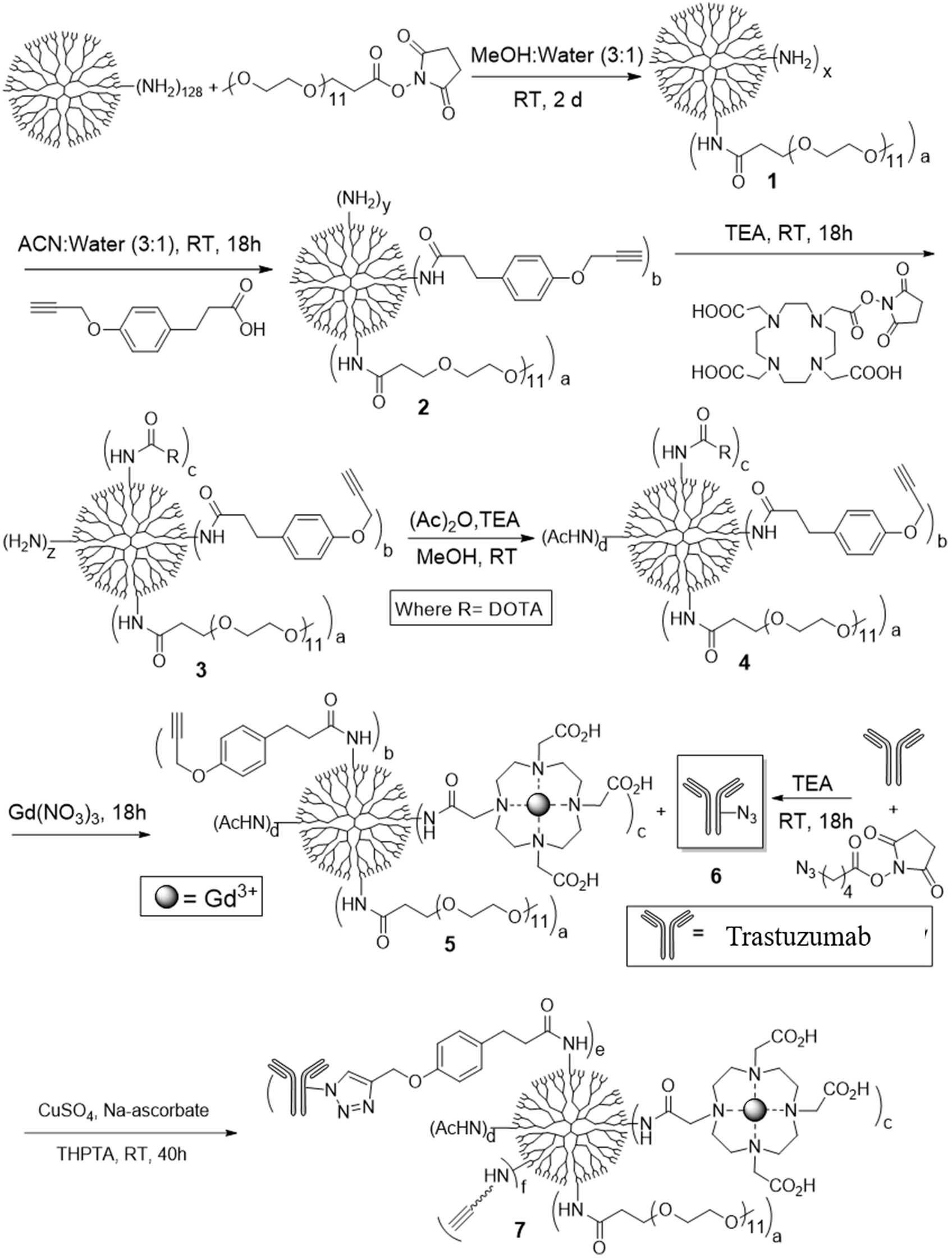

Supplement: Supplementary file 1 — Additional file 1: Figure S1. Preparation of G5-Gd-Trastuzumab (7) and G5-Gd (5). [file 12951_2020_695_MOESM1_ESM.tif]

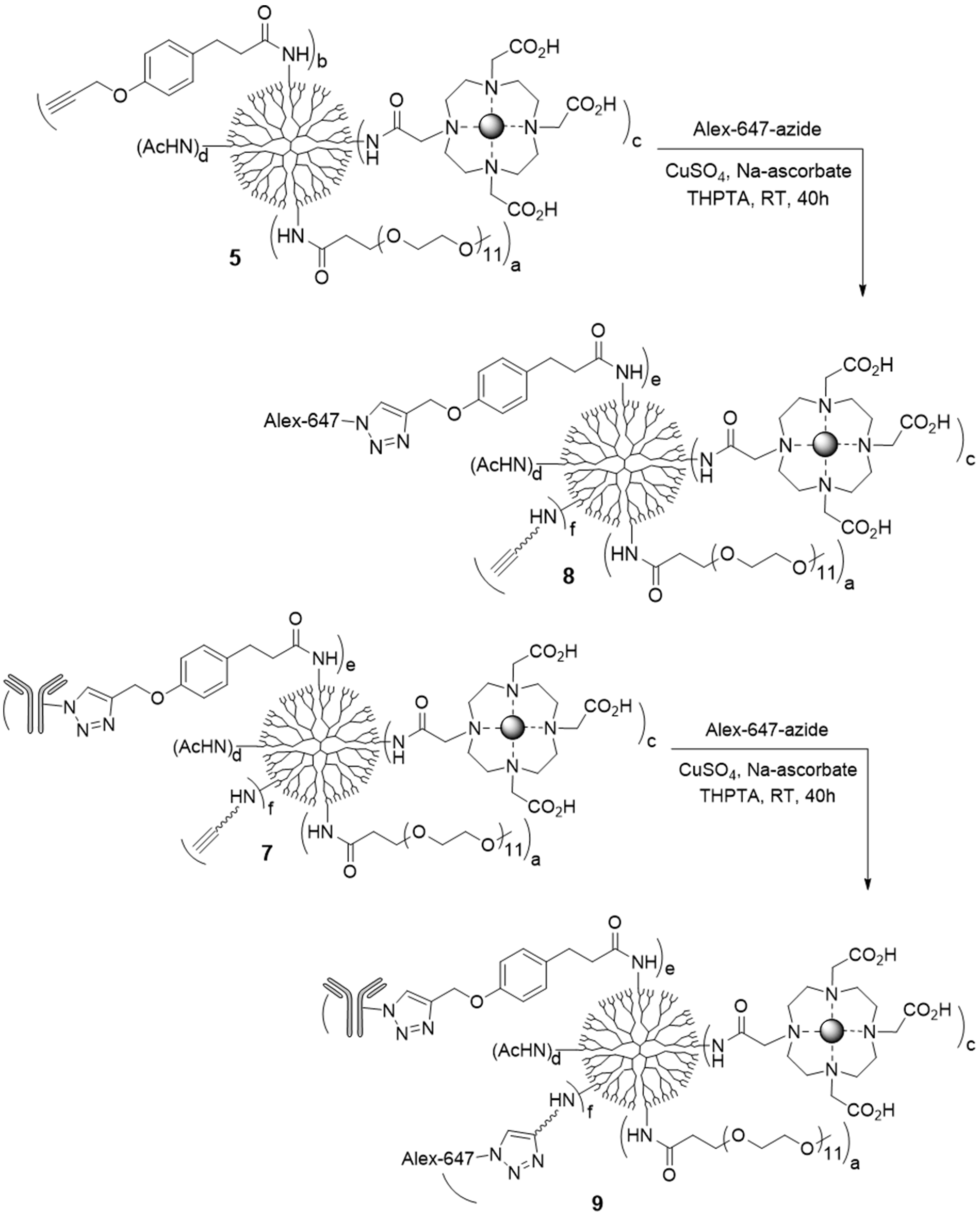

Supplement: Supplementary file 2 — Additional file 2: Figure S2. Preparation of G5-Gd-Trastuzumab-AF647 (9) and G5-Gd-AF647 (8), from G5-Gd-Trastuzumab (7) and G5-Gd (5). [file 12951_2020_695_MOESM2_ESM.tif]

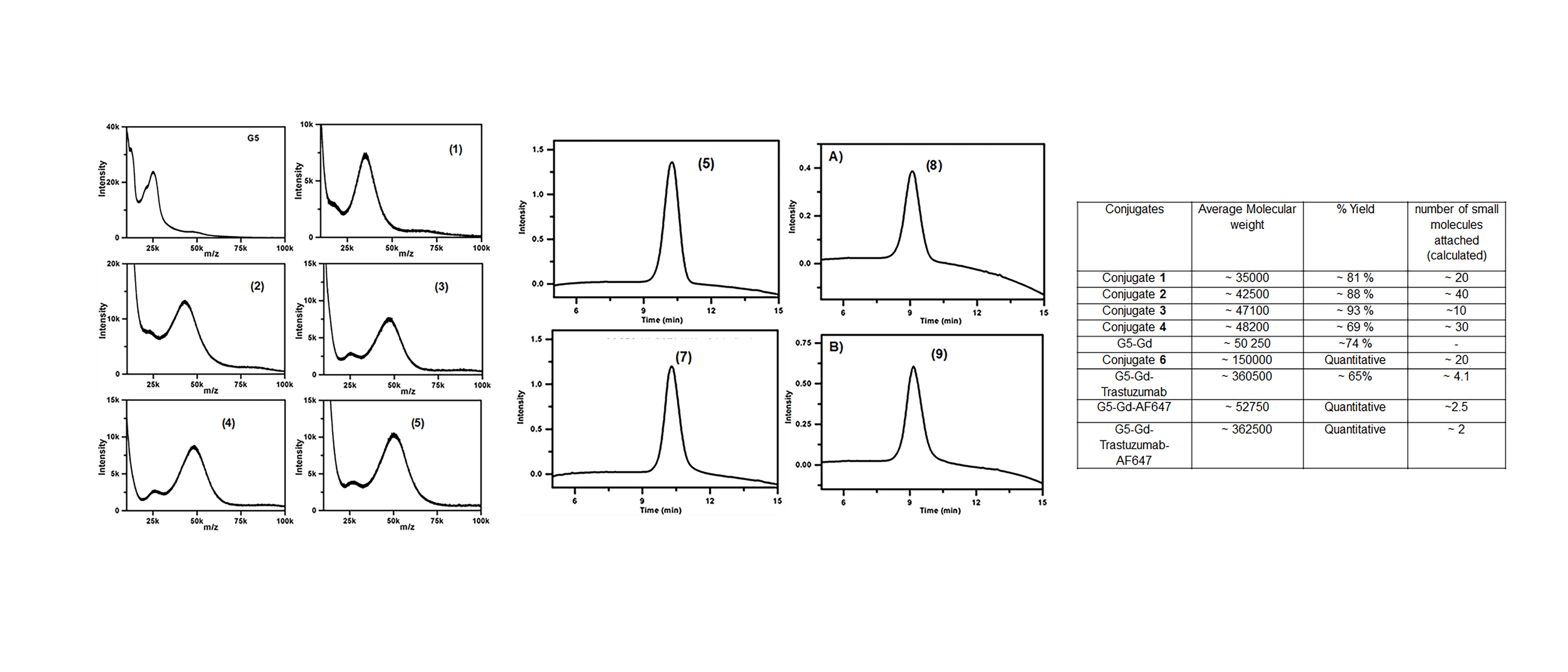

Supplement: Supplementary file 3 — Additional file 3: Figure S3. MALDI, UPLC, and synthesis information. [file 12951_2020_695_MOESM3_ESM.tif]

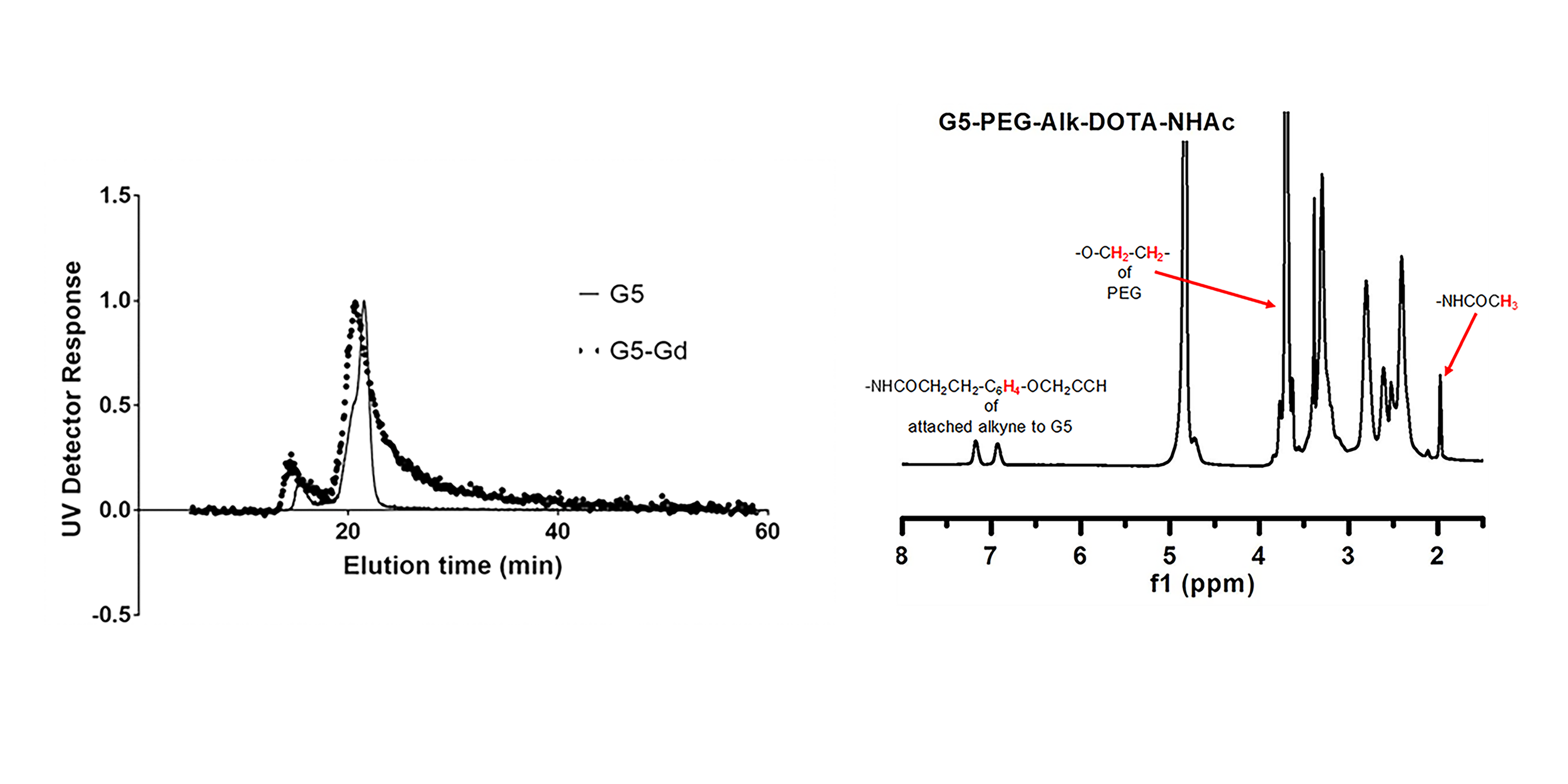

Supplement: Supplementary file 4 — Additional file 4: Figure S4. Size exclusion chromatography and NMR for G5-Gd. [file 12951_2020_695_MOESM4_ESM.tif]
